# High-Throughput Deconvolution of Native Protein Mass Spectrometry Imaging Data Sets for Mass Domain Analysis

**DOI:** 10.1021/acs.analchem.3c02616

**Published:** 2023-09-06

**Authors:** Oliver J. Hale, Helen J. Cooper, Michael T. Marty

**Affiliations:** † School of Biosciences, University of Birmingham, Edgbaston, Birmingham B15 2TT, U.K.; ‡ Department of Chemistry and Biochemistry and Bio5 Institute, University of Arizona, 1306 E University Blvd Tucson, Arizona 85721, United States

## Abstract

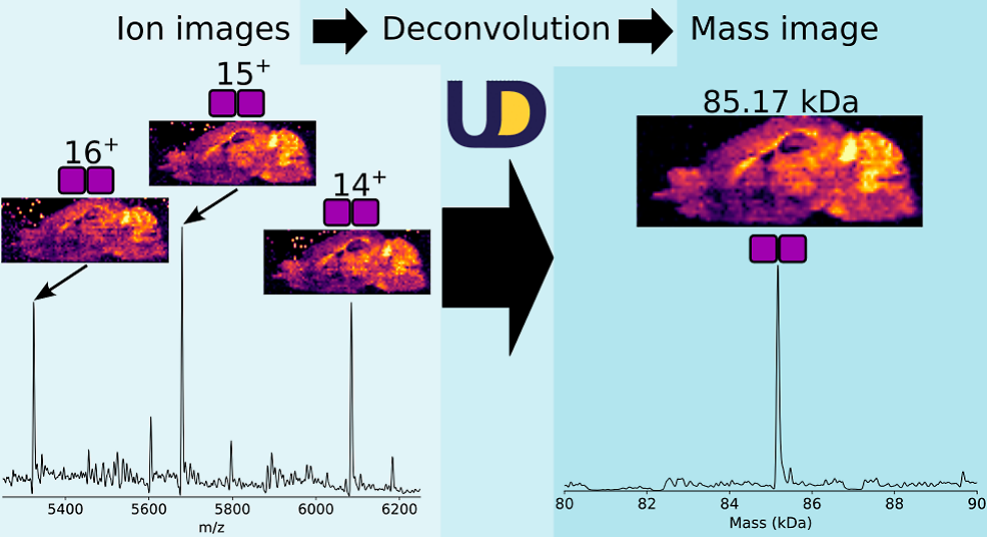

Protein mass spectrometry imaging (MSI) with electrospray-based ambient ionization techniques, such as nanospray desorption electrospray ionization (nano-DESI), generates data sets in which each pixel corresponds to a mass spectrum populated by peaks corresponding to multiply charged protein ions. Importantly, the signal associated with each protein is split among multiple charge states. These peaks can be transformed into the mass domain by spectral deconvolution. When proteins are imaged under native/non-denaturing conditions to retain non-covalent interactions, deconvolution is particularly valuable in helping interpret the data. To improve the acquisition speed, signal-to-noise ratio, and sensitivity, native MSI is usually performed using mass resolving powers that do not provide isotopic resolution, and conventional algorithms for deconvolution of lower-resolution data are not suitable for these large data sets. UniDec was originally developed to enable rapid deconvolution of complex protein mass spectra. Here, we developed an updated feature set harnessing the high-throughput module, MetaUniDec, to deconvolve each pixel of native MSI data sets and transform *m*/*z*-domain image files to the mass domain. New tools enable the reading, processing, and output of open format .imzML files for downstream analysis. Transformation of data into the mass domain also provides greater accessibility, with mass information readily interpretable by users of established protein biology tools such as sodium dodecyl sulfate polyacrylamide gel electrophoresis.

## Introduction

Analysis of the spatial distribution of biomolecules in tissue is an important aspect of life sciences research and involves a wide range of techniques.^1−4^ Mass spectrometry imaging (MSI) is routinely used to map the spatial distribution of metals^5^ and small molecules (e.g., metabolites and lipids^1,6^) with a variety of different sampling techniques. MSI of intact proteins is much less common due to the technical challenges of ionizing proteins directly from a substrate and manipulating large ions in the gas phase. Methods involving on-tissue proteolysis^7,8^ and targeted protein tags^7,9,10^ are alternatives that avoid analyzing intact protein ions and allow the distribution of proteins to be mapped, but information on proteoform distributions, complex stoichiometry, and non-covalent interactions with ligands is lost with these methods.

Native MSI of proteins under non-denaturing conditions is an emerging subfield, offering the ability to map protein complexes in an untargeted fashion. The method was initially demonstrated with liquid extraction surface analysis (LESA)^11^ and advanced using nanospray desorption electrospray ionization (nano-DESI).^12^ Both ion sources are electrospray ionization-based^13^ and generate multiply charged protein ions. Nano-DESI, in particular, has evolved to enable MSI of soluble and membrane protein complexes from biological tissues at moderate spatial resolutions.^14−16^

For protein ions, the signal is typically divided among multiple charge states. This effect is more pronounced with increasing protein mass, so it more adversely affects detection of larger proteins. One solution to address the splitting of a signal and the subsequent impact on signal quality is to combine all signals from one protein species in post-processing. Previously, we have generated ion images for each individual charge state *m*/*z* of proteoforms in a non-automated procedure and then summed the images using a MATLAB script to generate pseudo-mass domain images.^15^ This process is laborious and a barrier to wider adoption of native protein MSI. It also does not account for potential overlapping contributions of multiple analytes with different charges and masses to a single *m*/*z* value.

Another potential solution to the problem of multiple charge states is to deconvolve each pixel’s spectrum and assemble images from these deconvolved spectra. Deconvolution of protein mass spectrometry data combines the signal from all charge states to convert the data from the *m*/*z* domain into the mass domain, which is often easier to interpret. Deconvolution algorithms can also quantitatively separate overlapping peaks. A variety of algorithms have been developed that have been discussed previously.^17,18^ Many algorithms, especially those used in top-down proteomics applications,^18^ require isotopically resolved data, which is often lacking in native MSI. Algorithms capable of deconvolving non-isotopically resolved data tend to be slower or require more manual intervention,^17^ both of which are unsuitable for large scale imaging data sets. UniDec, which uses a Bayesian algorithm for deconvolution, has emerged as a powerful approach for deconvolution of lower resolution data due to its speed and ability to deconvolve complex data.^19^ Recent studies have expanded the application of UniDec to high-throughput analysis with either MetaUniDec^20^ or the UniDec Processing Pipeline.^21^

Here, we describe modifications to UniDec^19^ to enable per-pixel deconvolution of protein MSI data through the MetaUniDec module.^20^ A standard .imzML data file (*m*/*z*, position, and intensity dimensions) can be imported, processed, and then exported as a new .imzML (mass, position, and intensity dimensions) enabling mass domain image analysis in dedicated MSI software. We applied this new imaging deconvolution workflow to a newly generated imaging data set from mouse brain and a previously published imaging data set from sheep eye lens. The images produced using MetaUniDec were statistically similar to those produced by the non-automated method, as assessed by their cosine similarity.^22^ Our examples show the potential of automated deconvolution for analysis of protein MSI data sets.

## Experimental Section

### Existing Data Sets

The eye lens data set has been published previously^14^ and is available at https://doi.org/10.25500/edata.bham.00000840 (open access) (see the folder for Figure 2).

### Materials

MS-grade water was purchased from Fisher Scientific (Loughborough, UK). HPLC-grade ammonium acetate was bought from J.T. Baker (Deventer, Netherlands). The detergent tetraethylene glycol monooctyl ether (C_8_E_4_) was bought from Sigma-Aldrich (Gillingham, UK). Mass spectrometer calibration was performed using FlexMix (Thermo Fisher, San Jose, CA). Nitrogen (>99.995%) and helium (>99.996%) gases used on the mass spectrometer were obtained from BOC (Guildford, UK).

### Animal Tissues

Fresh frozen brain from wild-type mice was the gift of Dr Richard Mead, University of Sheffield. The brain was sectioned in the sagittal plane to 10 μm thickness at −22 °C, thaw mounted onto glass microscope slides, and stored at −80 °C until analysis. Tissue sections were thawed but not washed or further prepared before analysis.

### Nano-DESI Mass Spectrometry of Mouse Brain

The mouse brain section was analyzed using a home-built nano-DESI ion source (described previously^16^) attached to an Orbitrap Eclipse mass spectrometer (Thermo Fisher, San Jose, USA) featuring the proton transfer charge reduction (PTCR), ETD, and HMR^n^ options. In-house developed software controlled the nano-DESI source, and the Orbitrap Eclipse was controlled by Tune 3.5 (Thermo). The solvent system was 200 mM aqueous ammonium acetate + C_8_E_4_ detergent at 0.5× critical micelle concentration and flowed at 2 μL/min. The spray voltage was optimized between 800 and 1300 V. The ion transfer tube temperature was 275 °C. The ion routing multipole pressure was set to 20 mTorr with nitrogen. Source dissociation voltage was set to 90 V. Source compensation scaling was 14%.

### PTCR MS Imaging

For targeted analysis of the creatine kinase homodimer, a PTCR MS^2^ method was used to generate a charge-reduced protein ion series from the isolated precursor *m*/*z*.^23^ We described a similar experiment previously for small protein complexes in liver tissue.^24^ Creatine kinase cations of *m*/*z* 5011 ± 150 (17^+^ charge state) were isolated in the linear ion trap (LIT) and stored in the center section of the high-pressure cell (HPC). The cation automatic gain control (AGC) target was 5 × 10^6^ charges with a maximum fill time of 2000 ms. Perfluoroperhydrophenanthrene (PFPP) anions were generated by an internal ion source, *m*/*z* selected in the quadrupole mass filter and collected in the front section of the LIT HPC for up to 200 ms (reagent ion AGC target 2 × 10^5^ charges). Cation and anion populations were then mixed for 15 ms, resulting in charge reduction of creatine kinase cations, which were then transmitted to the Orbitrap for *m*/*z* analysis. The Orbitrap resolution setting was 7500 (FWHM at *m*/*z* 200, transient length 16 ms).

### Data Processing

**Figure 1:**
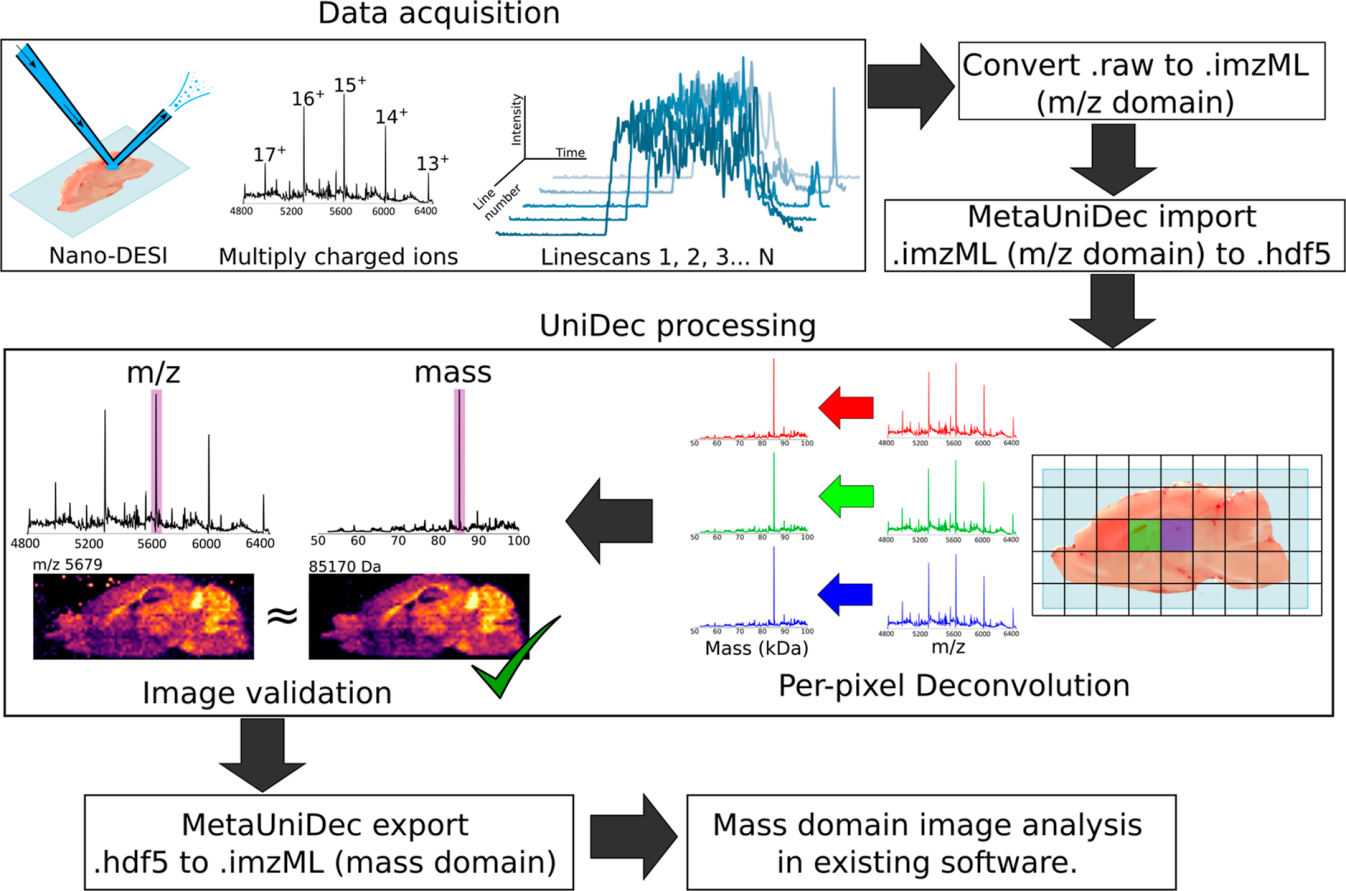
Data acquisition; data were acquired by nano-DESI. Raw data files were converted to an *m*/*z* domain .imzML file. The .imzML file was imported to MetaUniDec. UniDec processing; each pixel of the *m*/*z* domain .imzML file was deconvolved to the mass domain. The appearance of ion (*m*/*z*) and mass images was manually validated. For image analysis, the deconvolved data were exported as a new, mass domain .imzML file for analysis in dedicated MSI software, if desired.

The compiled program and source code for MetaUniDec are available online as part of the UniDec software package: https://github.com/michaelmarty/UniDec. In the version of UniDec used here (version 5.2.0), image processing functions can be accessed from the “Experimental” dropdown menu. An overview of the workflow is depicted in Figure 1.

Data processing was performed on a Dell Latitude 5500 laptop computer containing an Intel Core i7-8665U CPU (2.1 GHz base clock, 4 cores, 8 threads), 16 GB of 2400 MHz DDR4 RAM, and 1 TB NVMe solid state drive and running Windows 10 Enterprise N (version 21H2).

Nano-DESI line scans were converted from Thermo .raw files into a single, *m*/*z* domain .imzML file by Firefly (version 3.2.0.23, Prosolia Inc., Indianapolis, IN). Note: it was necessary to pass Firefly .imzML files through imzML Converter^25^ (version 1.3, University of Birmingham, UK) to fix a compatibility issue with the Python imzML reader, pyimzML (https://github.com/alexandrovteam/pyimzML), which is used by the import tool in MetaUniDec. The *m*/*z* domain .imzML files were imported into MetaUniDec, which converts them to the HDF5 format.^26^ Each spectrum is stored in the HDF5 file, as previously described,^20^ and position data are embedded as *x*, *y*, and *z* metadata for each spectrum. Per-pixel deconvolution was performed; settings used per data set can be found in the Supporting Information (Tables S1 and S2, Supporting Information). Deconvolution was validated by comparing the mass domain images to their component *m*/*z* images using the “Image Viewer” tool that was added into MetaUniDec. The deconvoluted data were then exported from UniDec as a new mass domain .imzML file for processing of images in dedicated MSI software. The *m*/*z* and mass domain .imzML files were viewed using MSiReader (v1.2, NC State University, Raleigh, NC).^27^ For comparison of composite ion images and mass images, ion images for each charge state of creatine kinase B-type were exported from MsiReader as MATLAB .fig files. Ion images for the four charge states were summed using a custom MATLAB script (available at https://github.com/coopergroup-massspec/sum_matlab_figures). The similarity of ion and mass images was scored using cosine similarity assessment included in the Image Viewer tool in MetaUniDec, or by an external Python script (available at http://www.biosciences-labs.bham.ac.uk/cooper/software.php.^22^

### Terminology

For the purposes of discussion, the following definitions are used to describe different image types:

- ion image: an image composed of a single *m*/*z* of a proteoform from an *m*/*z* domain .imzML file.
- composite ion image: a pseudo-mass domain image composed of the summed data from multiple ion images generated in a non-automated procedure, each representing different charge states of the same proteoform, from a *m*/*z* domain .imzML file.
- mass image: an image produced with a mass value from a mass domain .imzML file produced by deconvolution, such as through UniDec.

## Results and Discussion

### Targeted Imaging with PTCR

PTCR MS^2^ imaging of the creatine kinase B-type (CKB) homodimer in mouse brain was used to test the UniDec imaging workflow. CKB (calculated mass 85.165 kDa) was identified by nano-DESI native top-down MS, see Figure S1, Supporting Information. PTCR MS^2^ generates charge reduced product ion series from precursor ions within the isolated *m*/*z* range.^23^ PTCR product ion signals confer charge information without the need for isotopic resolution and can be deconvolved. PTCR also reduces underlying chemical noise and overlapping protein signals that differ in the charge state to the intended precursor ions.

**Figure 2:**
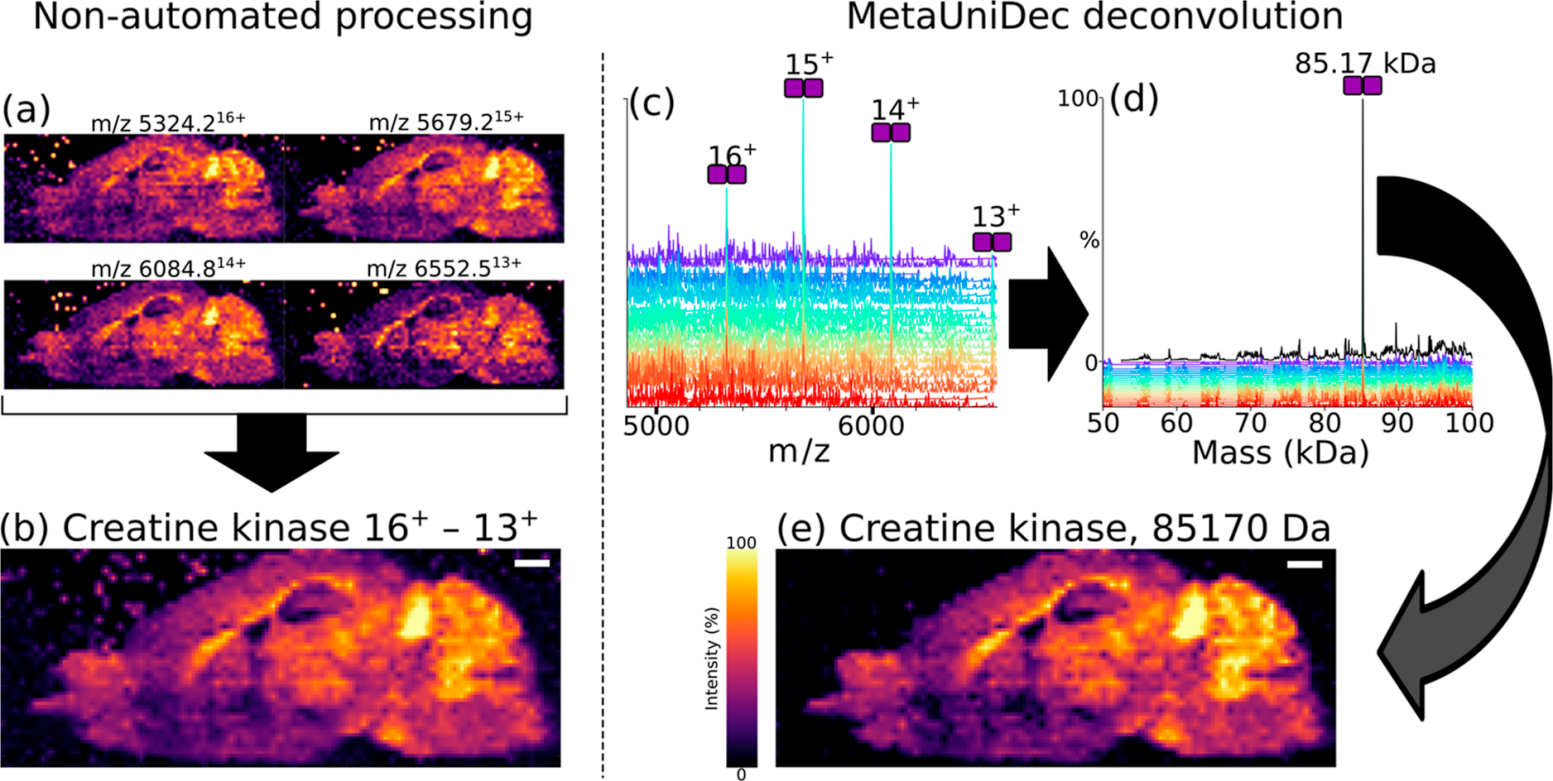
(a) Ion images for four charge states (16^+^–13^+^) of the creatine kinase homodimer produced by PTCR of the 17^+^ charge state (*m*/*z* 5011). (c) *m*/*z* and (d) mass spectra displayed for a subset of pixels in the data set. Colored traces represent a subset of the individual pixels. Deconvolved mass spectrum for the entire image data set is shown by the black trace. (e) Mass image for creatine kinase (85,170 ± 10 Da) produced from the output mass domain .imzML file.

Intact ions of the CKB homodimer were isolated at *m*/*z* 5011^17+^ ± 150 and subjected to the PTCR ion–ion reaction, generating 16^+^–13^+^ charge-reduced product ions, see Figure S2, Supporting Information. Ion images were generated for each product ion charge state (Figure 2a), using MSiReader to process the *m*/*z* domain file and were summed into the composite ion image using an existing MATLAB script (charge states 16^+^–13^+^, Figure 2b; script available from https://github.com/coopergroup-massspec/sum_matlab_figures). UniDec deconvolution of the *m*/*z* data (Figure 2c) generated the mass domain data with one abundant mass peak (85,170 Da, Figure 2d). Comparison of the composite ion image and the mass image for 85,170 ± 10 Da shows areas of the greatest (e.g., cerebellum) and weakest (e.g., olfactory bulb) relative signal intensity were well correlated between the two images (Figure 2b,e). The cosine similarity of the composite ion image and mass image is 98.1%, indicating high statistical similarity. The cosine similarity scores for CKB ion images were also assessed (all >94% similar to the mass image) and are shown in Figure S3, Supporting Information. CKB is known to have high expression levels in the cerebellum, in agreement with the images shown here.^28^ These data validate the UniDec imaging workflow for generating mass images of targeted proteins with minimal user intervention.

### Untargeted Analysis—Eye Lens Protein Complexes

A major appeal of MSI is its functionality as an untargeted imaging technique via broadband *m*/*z* analysis. For intact proteins, mass spectra in full scan modes are more challenging to deconvolute than a single charge state distribution from, e.g., PTCR experiments.

**Figure 3:**
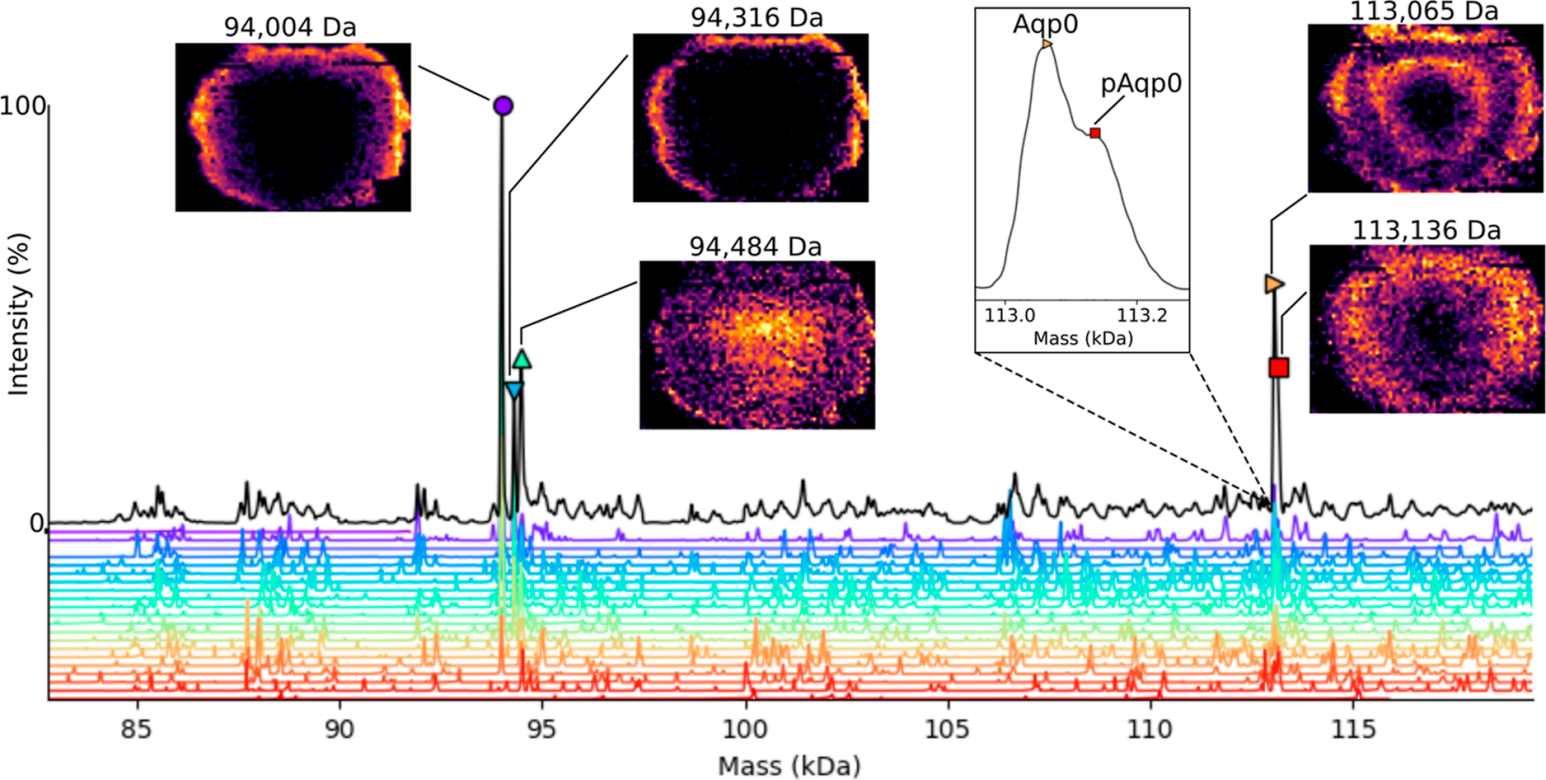
Deconvolved mass spectrum and mass images from the eye lens data set. The black trace depicts the deconvolved mass spectrum for the entire data set, while colored lines show deconvolved spectra from a subset of individual pixels. Mass images generated for each peak are depicted. 94,004 Da; beta-crystallin B2/B2/A4/A1 heterotetramer, 94,316 Da; unknown, 94,484 Da beta-B2-crystallin homotetramer with two ligands, 113,065 Da; Aqp0, and 113,136 Da; pAqp0. The inset spectrum shows the peak for Aqp0 and the peak shoulder assigned to pAqp0.

We applied the MetaUniDec workflow to the analysis of a previously published data set where we imaged protein complexes in the eye lens.^14^ Per-pixel deconvolution resolved peaks for tetrameric crystallin complexes and the tetrameric membrane protein assembly Aqp0 (Figure 3). Mass images for these complexes are comparable to the ion images and composite ion images we reported previously. Specifically, the cosine similarity for the Aqp0 mass image and Aqp0 composite image is 97.1%. We, and others, have also reported tetrameric Aqp0 featuring a single phosphorylated subunit (pAqp0), and it is also observable by per-pixel deconvolution here.^14,29^ The calculated mass difference between the Aqp0 tetramer and pAqp0 is approximately 80 Da. Signals for these proteoforms were not baseline resolved in the imaging data set, using an Orbitrap nominal resolution of 7500 (FWHM at *m*/*z* 200). We performed a separate experiment using a higher Orbitrap resolving power (30,000 FWHM at *m*/*z* 200) to confirm the mass difference in the original publication.^14^ In MetaUniDec, pAqp0 appears as a shoulder on the Aqp0 peak in the deconvoluted spectrum that is around 71 Da heavier (Figure 3 inset), and mass images for both proteoforms have comparable distributions to those produced by the non-automated method.^14^ The ability to deconvolve PTMs from a protein exceeding 100 kDa directly from the imaging mass spectra is promising, and we expect that continued developments in in situ native MS will further improve the technique.

**Figure 4:**
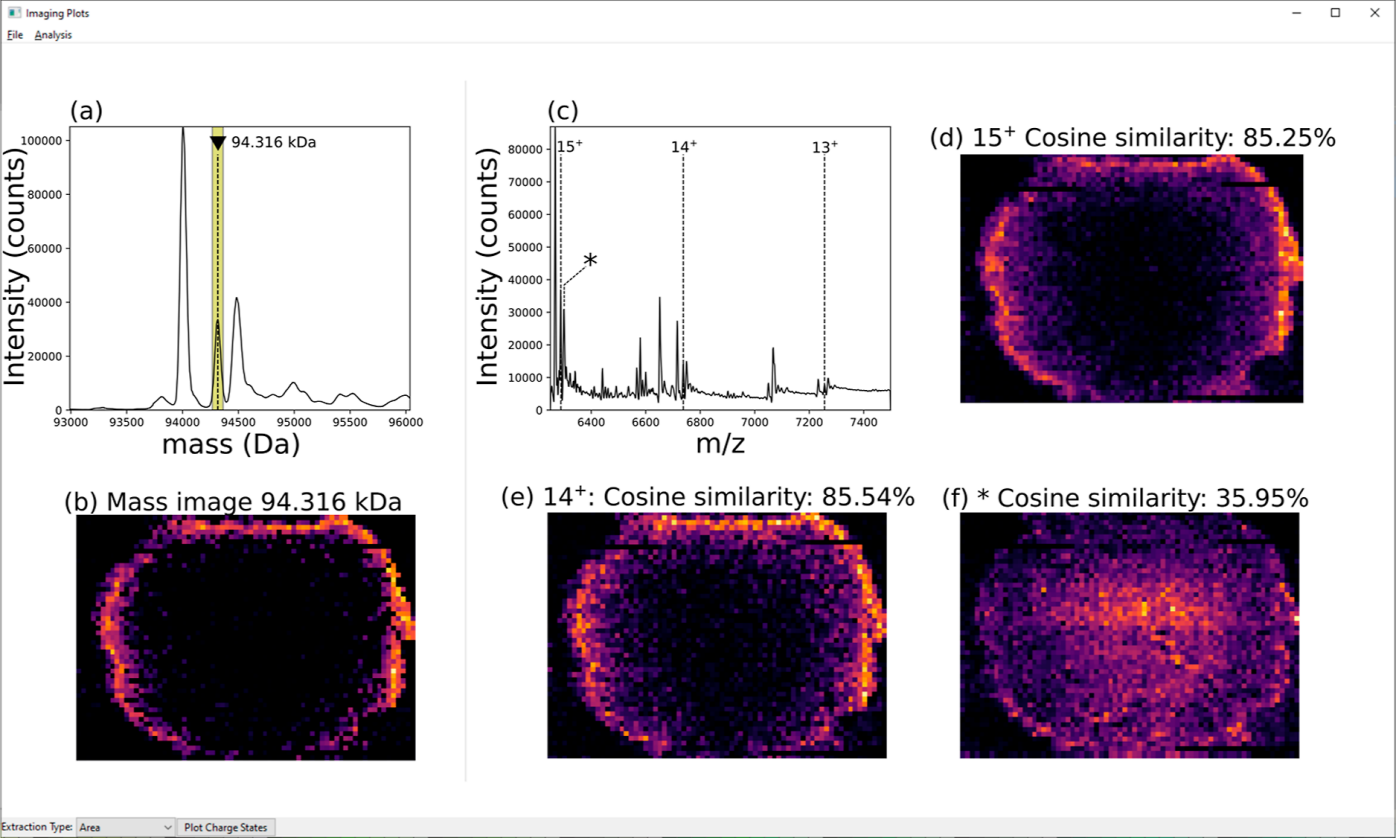
Panels showing functionality of the UniDec Imaging Viewer. (a) Peak at mass 94,316 Da selected in the deconvolved spectrum. (b) Mass image for MW 94,316 Da generated by selection of the peak in (a). (c) Sum mass spectrum (*m*/*z*) for the data set showing labeled charge states of the selected protein in (a,b). (d) Ion image for *m*/*z* 6288^15+^; 85.25% similar to the mass image. (e) Ion image for *m*/*z* 6738^14+^; 85.54% similar to the mass image. (f) Ion image for *m*/*z* 6299^15+^; 35.95% similar to the mass image. Note: the figure is a composite of multiple Image Viewer windows; only one *m*/*z* spectrum image and one ion image may be viewed at once.

MetaUniDec deconvolution of the eye lens imaging data set revealed a protein of 94.3 kDa that we did not investigate previously.^14^ The mass suggests that it is another tetramer composed of beta-crystallin isoforms. We validated the mass deconvolution by viewing each component ion image in MetaUniDec’s “Image Viewer” tool (Figure 4), currently accessed by selecting “Experimental → Open Imaging Viewer” from the toolbar. The Image Viewer enables mass and *m*/*z* images to be compared using interactive plots. Right-clicking the deconvoluted mass peak generates its mass image. Charge states composing the deconvoluted image can be viewed in the *m*/*z* spectrum. Right-clicking a *m*/*z* peak generates its ion image. The cosine similarity of mass and ion images can be calculated by selecting “Analysis” in the toolbar and selecting “Cosine Similarity mass vs *m*/*z*”. The score is displayed in the Image Viewer window and the UniDec console and allows the user to assess the similarity of ion images included in deconvolution. Importantly, ion images with differing spatial distributions are likely not signals for the same proteoform.

To demonstrate validation of the peaks, we selected the unknown peak (94,316 Da) from the deconvolved spectrum for the crystallin tetramer mass range (Figure 4a), and we generated the mass image corresponding to the selected peak (Figure 4b). The *m*/*z* spectrum was labeled for the charge states of the protein and an adjacent, unrelated peak (*). Ion images were extracted for the 15^+^ (Figure 4d) and 14^+^ (Figure 4e) charge states. Both ion images closely resemble the mass image as scored by their cosine similarity (15^+^; 85.25% and 14^+^; 85.54%). The scores indicate that each is similar to the mass image and is correctly assigned to the same proteoform. The image for the adjacent peak (Figure 4f; 35.95%) serves as an example of a low similarity image that is not part of the selected ion series identified by the deconvolution. Instead, this ion is the 15^+^ charge state of the 94.5 kDa crystallin complex in Figure 3.^14^

Note, due to differences between experiment conditions and spectral quality, it is most useful to establish score cutoffs on a per-experiment basis. The magnitude of scores for correctly assigned ions should tend toward 100%, and differences between each contributing ion image should be minimal. In our testing, scores above 80% were reliable indicators of similarity (see Figure S4, Supporting Information). Noise and proteins with differing spatial distributions scored lower (<80%). However, the exact score cutoff may be different between experiments, so positive and negative control comparisons like these should be applied within experiment data sets. Specifically, images of low similarity may still have a relatively high score magnitude because the glass slide pixels have a low signal and are essentially identical at all *m*/*z*. Thus, we would expect that images with a higher proportion of empty slide pixels would need higher score cutoffs.

### Limitations

The main limitation of the current workflow comes from the data. MSI under native conditions currently suffers from low absolute signal intensity and high non-specific background, particularly at >*m*/*z* 4000 on our system, meaning that some imaging data sets will not produce clear deconvolution results. Noise will contribute to deconvolution artifacts and false protein mass determination. Finally, the current method requires a single set of deconvolution parameters for the entire data set. If a specific pixel range needed a mass or *m*/*z* range that was different from the rest of the pixels, deconvolution would have to be repeated with different settings for all pixels.

As such, a level of manual review is still necessary. Manual review will likely remain important even with higher data quality as we expect that the number of protein signals within the data would also increase. However, the automation of mass image generation with UniDec reduces the amount of manual intervention needed and empowers the user to quickly review key features.

Computer resources (processing time, memory usage, and storage) may also be a barrier in some situations. The eye lens data set contains around 3000 pixels and required about 4 min of processing for spectral deconvolution, which corresponds to a rate of around 12 spectra per second. High spatial resolution MSI experiments may contain millions of pixels.^30,31^ As higher spatial resolving powers become available, or if imaging of large tissue sections is required, then processing on local computer resources may be impractical. Fortunately, a Docker container is available (https://hub.docker.com/r/michaeltmarty/unidec) for deployment on high-performance computing or cloud infrastructure. Because UniDec is open-source and has a Python API, it can be easily incorporated into custom scripts for automated processing and analysis. At a rate of 12 spectra per second, it should be possible to process a million spectra in a day. Further computational advances could also speed up analysis by streamlining hard drive reading/writing and optimizing parallel processing for large scale spectra. Because UniDec provides a free and open-source platform for deconvolution of imaging data sets, users can customize and optimize this platform for their own unique applications and infrastructure.

## Conclusions

Accessible data analysis tools are an important aspect for adoption of a fledgling analytical method, and the native protein MS community have called for development of software tools that match the performance of contemporary instrumentation.^32^ Imaging tools and workflows added to MetaUniDec provide automation of previously arduous data analysis steps for native protein MSI data sets and transform the data to be more readily interpretable by non-specialists. We have demonstrated the utility of MetaUniDec for a simple data set (creatine kinase in mouse brain) and a complex data set (eye lens). Although the data quality currently causes some limitations, we expect these to reduce in impact as the imaging methods and instrumentation evolve. We believe that the availability of this free and open-source software will enable others to adopt protein MSI using ESI-based ion sources where data processing was previously a barrier to entry.

## Supporting Information Available

Tables with UniDec processing parameters and sequence ions and figures of the MS2 spectrum of the CKB homodimer, PTCR spectrum of CKB, image viewer analysis of CKB images, and a comparison of mass and ion images from eye lens data (PDF)
