# Supplementary material for: High-Throughput Deconvolution of Native Protein Mass Spectrometry Imaging Data Sets for Mass Domain Analysis
Source: Anal Chem. 2023 Sep 6;95(37):14009–15. doi: 10.1021/acs.analchem.3c02616 (PMC10515104; doi:10.1021/acs.analchem.3c02616)
Supplement: Supplementary file 1 — ac3c02616_si_001.pdf [file ac3c02616_si_001.pdf]

Supporting Information for:

High-throughput deconvolution of native protein mass spectrometry imaging datasets for mass domain analysis.

Oliver J. Hale[1], Helen J. Cooper*[1], Michael T. Marty*[2]

1: School of Biosciences, University of Birmingham, Edgbaston, Birmingham, B15 2TT, UK.

2: Department of Chemistry and Biochemistry and Bio5 Institute, University of Arizona, 1306 E University Blvd, 85721, Tucson, AZ, USA

* h.j.cooper@bham.ac.uk and mtmarty@arizona.edu

Table S1: UniDec processing parameters for the mouse brain dataset 2
Table S2: UniDec processing parameters for the eye lens dataset 3
Table S3: Sequence ions for CKB. 3

Figure S1: Nano-DESI-HCD MS^2 spectrum of creatine kinase B homodimer 4
Figure S2: Representative PTCR MS^2 spectrum for CKB. 5
Figure S3: Image Viewer analysis of CKB images. 5
Figure S4: Comparison of mass and ion images from eye lens........ 6

Table S1: UniDec processing parameters for the mouse brain dataset. Unspecified values were left at the default value.

Parameter	Value
**Data processing**	
m/z	4865 to 6574
Background subtraction	0
Bin Every	0
Data reduction (%)	0
Intensity threshold	0
Normalize data	Unchecked
**UniDec Parameters**	
Charge range	5 to 25
Mass range (Da)	50,000 to 100,000
Sample mass every (Da)	10
**Quick controls**	
Smooth charge states distributions	Checked
Use automatic m/z peak width	Checked
Smooth nearby points	Some
Suppress artifacts	None
Mass differences (Da)	Unchecked
**Peak selection, extraction, and plotting**	
Picking range (Da)	100
Picking threshold	0.2
Peak normalization	Max
How to extract peaks	Height
Extraction window	0
Extraction threshold	10

Table S2: UniDec processing parameters for the eye lens dataset. Unspecified values were left at the default value.

Parameter	Value
**Data processing**	
m/z	6250 to 7500
Background subtraction	0
Bin Every	0
Data reduction (%)	0
Intensity threshold	5
Normalize data	Unchecked
**UniDec Parameters**	
Charge range	12 to 18
Mass range (Da)	80,000 to 120,000
Sample mass every (Da)	1
**Quick controls**	
Smooth charge states distributions	Checked
Use automatic m/z peak width	Checked
Smooth nearby points	Some
Suppress artifacts	Some
Mass differences (Da)	Unchecked
**Peak selection, extraction, and plotting**	
Picking range (Da)	10
Picking threshold	0.2
Peak normalization	Max
How to extract peaks	Height
Extraction window	5
Extraction threshold	10

Table S3: Sequence ions for CKB from Figure S1.

Ion	z	Theoretical Mass (Da)	Observed Mass (Da)	Mass Difference (ppm)
b55	5	6167.9550	6167.9260	-4.7
b56	5	6281.0390	6281.0630	3.8
b57	5	6409.0975	6409.0960	-0.2
b58	5	6510.1450	6510.1490	0.6
y98	7	10638.6917	10638.6875	-0.4
y104	7	11289.0015	11288.9700	-2.8
y107	7	11636.1951	11636.1311	-5.5
y107	6	11636.1972	11636.1714	-2.2

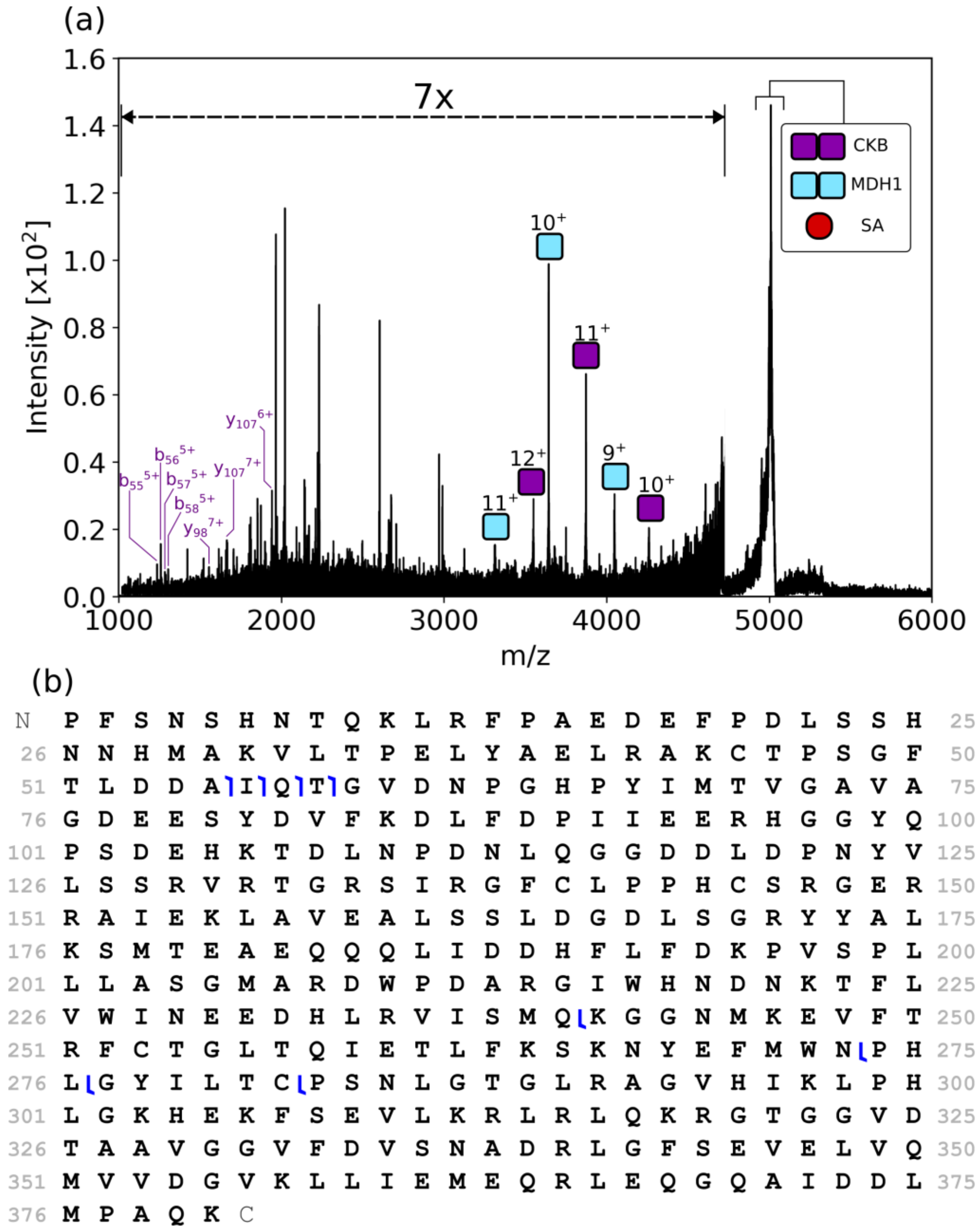

Figure S1: (a) nano-DESI-HCD MS^2 spectrum of creatine kinase B homodimer (~m/z 5011^{17+}). Homodimers are dissociated to monomers (deconvolved mass = 42,583 Da, calculated mass = 42,582.7 Da) and sequence fragments (purple) with HCD normalised collision energy 35 – 40%. Overlapping proteins include MDH1 homodimer (pale blue) and serum albumin (red). Ion trap Isolation window = m/z 5011 +/- 7.5. (b) sequence ions annotated on the amino acid sequence of CKB. Additional details are provided in Table S3 above.

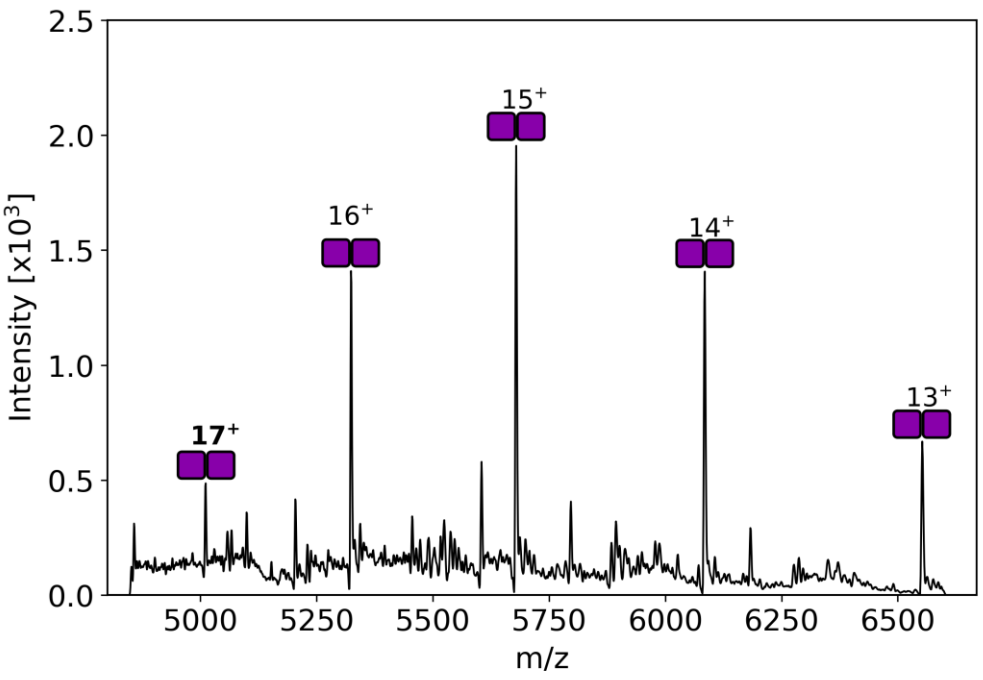

Figure S2: Representative PTCR MS2 spectrum for CKB. Isolation window m/z 5011 +/- 150. Four charge-reduced product ion charge states (16+–13+) were detected. Generated from 226 on-tissue orbitrap scans from Line 14 of the PTCR image.

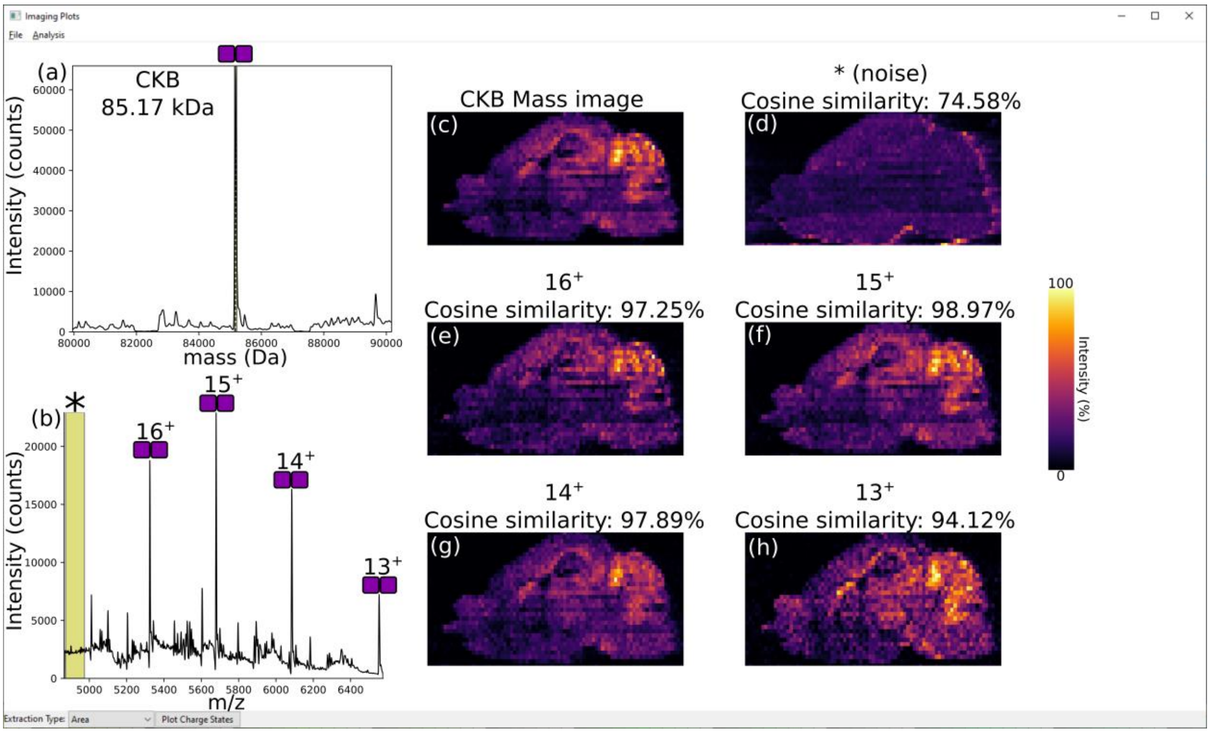

Figure S3: Image Viewer analysis of CKB images. (a) Deconvolved mass spectrum showing CKB peak. (b) PTCR mass spectrum showing four charge states of CKB. The asterisk indicates a region of noise. (c) Mass image for CKB in (a). (d) Ion image for the noise region (*) in (b) which is only 74.58% similar to the mass image. CKB charge state ion images have a high cosine similarity to the CKB mass image: (e) 16+; 97.25%, (f) 15+; 98.97%, (g) 14+, 97.89% and (h) 13+; 94.12%. Note: The figure is a composite of multiple Image Viewer windows; only one m/z spectrum and ion image may be viewed at once.

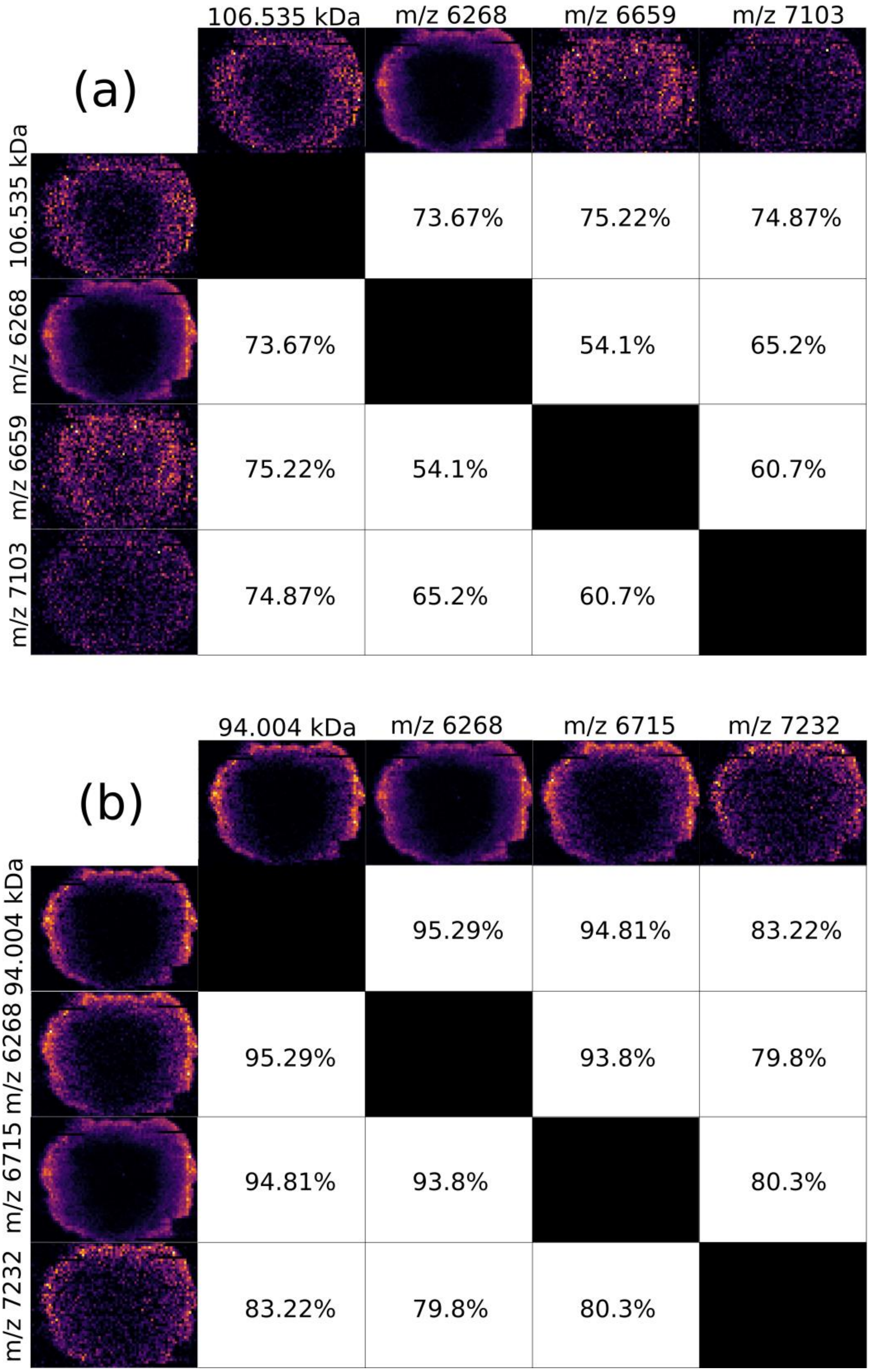

Figure S4: Comparison of mass and ion images. (a) A rejected deconvolved mass, 106.535 kDa. The mass image for 106.535 kDa is composed of ion mages that are less than 80% similar to the mass images. Each ion image is also dissimilar to the others. This assessment indicates incorrect assignment. (b) An acceptable deconvolved mass, 94.004 kDa (known to be a β-B2-crystallin tetrameric complex). Each ion image is more than 80% similar to the mass image. Ion images are also similar to one another. This assessment indicates correct assignment. Also note that m/z 6268 is included in both mass images, but only scores highly for the 94.004 kDa image.
